# Quantifying the age structure of free‐ranging delphinid populations: Testing the accuracy of Unoccupied Aerial System photogrammetry

**DOI:** 10.1002/ece3.10082

**Published:** 2023-06-26

**Authors:** Fabien Vivier, Randall S. Wells, Marie C. Hill, Kymberly M. Yano, Amanda L. Bradford, Eva M. Leunissen, Aude Pacini, Cormac G. Booth, Julie Rocho‐Levine, Jens J. Currie, Philip T. Patton, Lars Bejder

**Affiliations:** ^1^ Marine Mammal Research Program Hawaiʻi Institute of Marine Biology University of Hawaiʻi at Mānoa Mānoa Hawaiʻi USA; ^2^ Chicago Zoological Society's Sarasota Dolphin Research Program c/o Mote Marine Laboratory Sarasota Florida USA; ^3^ Cooperative Institute for Marine and Atmospheric Research Research Corporation of the University of Hawaiʻi Honolulu Hawaiʻi USA; ^4^ Pacific Islands Fisheries Science Center NOAA Fisheries Honolulu Hawaiʻi USA; ^5^ Department of Marine Science University of Otago Dunedin New Zealand; ^6^ SMRU Consulting Scottish Oceans Institute University of St Andrews St Andrews UK; ^7^ Dolphin Quest Oʻahu Honolulu Hawaiʻi USA; ^8^ Pacific Whale Foundation Wailuku Hawaiʻi USA; ^9^ Zoophysiology Department of Bioscience Aarhus University Aarhus Denmark

**Keywords:** conservation, demography, dolphin, drone, photogrammetry, population, precision, UAS

## Abstract

Understanding the population health status of long‐lived and slow‐reproducing species is critical for their management. However, it can take decades with traditional monitoring techniques to detect population‐level changes in demographic parameters. Early detection of the effects of environmental and anthropogenic stressors on vital rates would aid in forecasting changes in population dynamics and therefore inform management efforts. Changes in vital rates strongly correlate with deviations in population growth, highlighting the need for novel approaches that can provide early warning signs of population decline (e.g., changes in age structure). We tested a novel and frequentist approach, using Unoccupied Aerial System (UAS) photogrammetry, to assess the population age structure of small delphinids. First, we measured the precision and accuracy of UAS photogrammetry in estimating total body length (TL) of trained bottlenose dolphins (*Tursiops truncatus*). Using a log‐transformed linear model, we estimated TL using the blowhole to dorsal fin distance (BHDF) for surfacing animals. To test the performance of UAS photogrammetry to age‐classify individuals, we then used length measurements from a 35‐year dataset from a free‐ranging bottlenose dolphin community to simulate UAS estimates of BHDF and TL. We tested five age classifiers and determined where young individuals (<10 years) were assigned when misclassified. Finally, we tested whether UAS‐simulated BHDF only or the associated TL estimates provided better classifications. TL of surfacing dolphins was overestimated by 3.3% ±3.1% based on UAS‐estimated BHDF. Our age classifiers performed best in predicting age‐class when using broader and fewer (two and three) age‐class bins with ~80% and ~72% assignment performance, respectively. Overall, 72.5%–93% of the individuals were correctly classified within 2 years of their actual age‐class bin. Similar classification performances were obtained using both proxies. UAS photogrammetry is a non‐invasive, inexpensive, and effective method to estimate TL and age‐class of free‐swimming dolphins. UAS photogrammetry can facilitate the detection of early signs of population changes, which can provide important insights for timely management decisions.

## Graphical abstract

Here, we use Unoccupied Aerial System (UAS) photogrammetry to develop a length‐based method of estimating the age‐class of free‐ranging delphinids. First, we measure the accuracy and precision of UAS photogrammetry in estimating the total body length (TL) and the distance between the blowhole and the dorsal fin insertion (BHDF) of trained bottlenose dolphins (*Tursiops truncatus*). Then, we test the performance of UAS photogrammetry in estimating the age‐class (based on TL and BHDF) of free‐ranging bottlenose dolphins. Our findings suggest that UAS photogrammetry is an effective method to estimate TL and age‐class of free‐swimming dolphins. This method can facilitate the detection of early signs of population changes, which can provide important insights for timely management decisions. Right photograph taken under research permit NOAA‐PIFSC 21476.

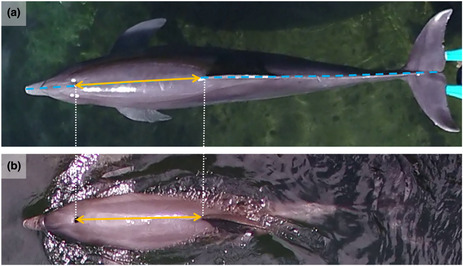

## 1 INTRODUCTION

The ability to monitor the health status and dynamics and detect trends of free‐ranging populations is critical for the effective management of long‐lived and slow‐reproducing species (Holmes & York, 2003; Jackson et al., 2020). For marine mammals, anthropogenic and environmental stressors can affect individual health and vital rates (e.g., fertility and survival; Pirotta et al., 2019) and subsequently cause population‐level impacts (e.g., habitat shift, and abundance decline; Pirotta et al., 2015; Senigaglia et al., 2016). A decline in population abundance and/or changes in vital rates can provide an early warning for the sustainability of a population. Therefore, early detection of stressor effects on individuals could help forecast potential impacts at the population level.

Cetacean population sizes are typically estimated via line‐transect or mark‐recapture surveys (e.g., Dawson et al., 2004; Evans & Hammond, 2004; Wade & Gerrodette, 1993). However, physiological and behavioral changes take time to manifest into changes in health and vital rates and, in turn, population size (Maxwell & Jennings, 2005; Symons et al., 2018; Taylor et al., 2007). Consequently, these traditional techniques often have a limited statistical power to estimate a population trend or detect a change in the trend (Taylor et al., 2007). Thus, relying only on abundance estimation to monitor population dynamics can inhibit timely conservation and management actions (Taylor & Gerrodette, 1993; Thompson et al., 2000; Turvey et al., 2007). Furthermore, the typical frequency of surveys and imprecision of abundance estimates may fail to detect precipitous declines in abundance (Taylor et al., 2007), highlighting the need for alternative techniques to help detect early warning signs of population declines.

Population dynamics are a function of key parameters, such as population growth and age structure (Clark et al., 2000; Jackson et al., 2020), which are function of vital rates (Ozgul et al., 2010) and environmental factors (e.g., Pardo et al., 2013; Weimerskirch, 2018). A stable age distribution is an indicator of population health, that is, the population contains a fixed proportion of newborn, immature, and mature individuals (Gamelon et al., 2016), while deviances from this distribution would lead to either population growth or decline (Coulson et al., 2005; Jackson et al., 2020; Jones et al., 2018). Therefore, detecting changes in the age structure of a population may provide an early sign of future changes in abundance (Booth et al., 2020; Holmes & York, 2003; Reichert et al., 2016). Few studies have focused on estimating the age structure of cetaceans to monitor population health (Evans & Hindell, 2004; Guo et al., 2020; Pallin et al., 2022).

Non‐invasive technologies such as aerial photogrammetry using Unoccupied Aerial Systems (UASs or “drones”) have become common practice in baleen whale health monitoring studies (Bierlich, Hewitt, et al., 2021; Bierlich, Schick, et al., 2021; Christiansen et al., 2018, 2022; Dawson et al., 2017). To date, few studies have examined the performance of UAS photogrammetry to monitor the health of toothed whales (Cheney et al., 2022; Currie et al., 2021; Fearnbach et al., 2018). UAS photogrammetry allows for large groups of animals to be sampled with minimal effort (Booth et al., 2020), suggesting that UAS photogrammetry might be a suitable and cost‐effective tool to monitor changes in the age structure of delphinid populations.

The overall aim of this study was to use UAS photogrammetry to develop a length‐based method of estimating the age‐class of free‐ranging delphinids. First, we evaluated the precision (variation between measurements) and accuracy (consistency between the estimated and observed measurements) of UAS photogrammetry for measuring and estimating the total body length (TL) of bottlenose dolphins (*Tursiops truncatus*) under human care. Second, we tested whether individual bottlenose dolphins could be assigned to correct age‐classes from simulated UAS photogrammetry length estimates as a means of quantifying the age structure of a well‐studied, free‐ranging dolphin community. Findings are discussed in the context of providing rapid and important insights for timely management and conservation of cetacean populations.

## 2 MATERIALS AND METHODS

### 2.1 Facilities, study animals, and length measurements

**FIGURE 1:**
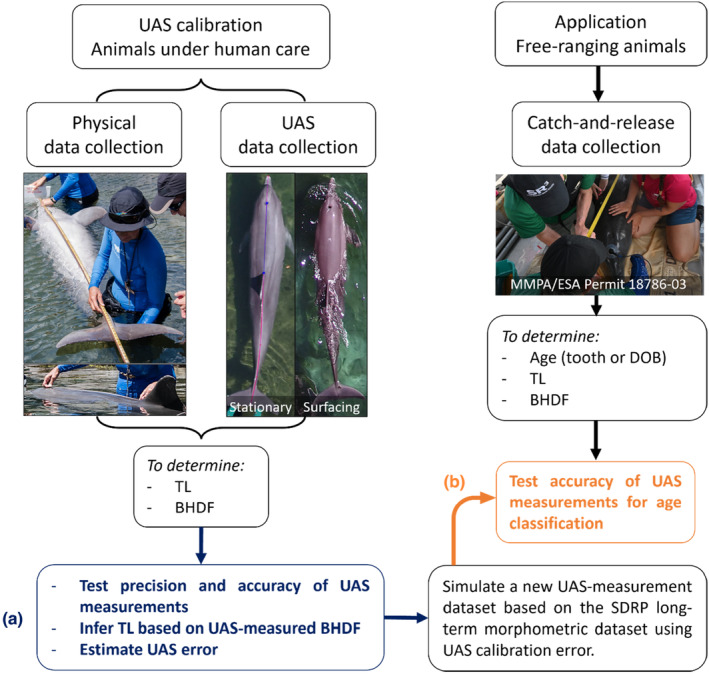
Workflow used to test the accuracy of Unoccupied Aerial System (UAS) photogrammetry in (a) estimating the total length (TL) of bottlenose dolphins under human care; and (b) inferring age‐class based on length and classifying individuals into age‐class bins using a long‐term dataset from the Sarasota Dolphin Research Program (SDRP), FL, USA. BHDF: Blowhole to Dorsal Fin Distance, DOB: Date of Birth. Abbreviations are defined in Table A1.

**FIGURE A1:**
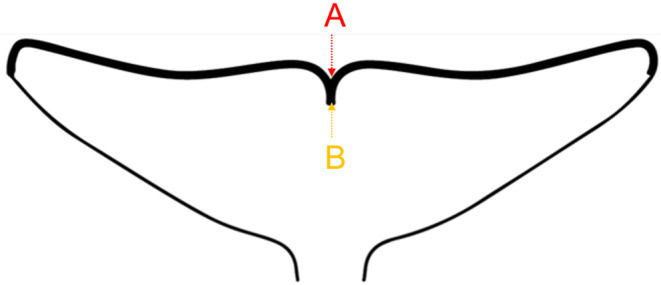
Location of the A) natural notch created by the overlap of the fluke lobes and B) notch where the fluke lobes separate. The natural notch created by the overlapping fluke lobes was used for total length measurement.

We physically measured TL (i.e., the tip of the rostrum to the tip of the natural notch created by the overlapping fluke lobes (Figure A1), hereafter referred to as the notch) and blowhole to dorsal fin (BHDF) for 18 bottlenose dolphins under human care at two facilities in Hawaiʻi, USA (Figure 1a). The distance from the center of the blowhole to the anterior insertion of the dorsal fin is an established proxy for TL in bottlenose dolphins (Cheney et al., 2018; van Aswegen et al., 2019). Six adult males ranging from 11.5 to 34.5 years of age (mean = 23.6 ± 7.9 years) at Dolphin Quest Oʻahu (DQO); HI, USA, were measured in June 2019. Six females and six males ranging from 4.0 to 49.0 years of age (mean = 17.4 ± 14.8 years) at Dolphin Quest Hawaiʻi (DQH); HI, USA, were measured in August–October 2019. The date of birth (DOB) of the 14 individuals born in facilities is known. The other four individuals (two males and two females) were born in the Gulf of Mexico. The age of these animals was based on the size that they were when collected. Dolphins were measured in a stationary and straight position for all measurements. TL was collected on the ventral side of the dolphin in an inverted position using a tape measure attached to a rigid PVC pipe. The base of the measuring pipe was placed onto a rigid plate aligned with the tip of the rostrum to allow for straight‐line measurements. BHDF measurements were made from the center of the blowhole to the insertion of the dorsal fin using a soft measuring tape. One measurement set (consisting of two to three replicates per measurement) was collected on the day or within a week of the UAS sampling (see below). To increase sample size, four to six additional replicates were collected within the next 7 months (total of 7–10 TL and BHDF measurements per animal). DQH measurements per animal were collected on the same day.

### 2.2 Length measurements via UAS photogrammetry

Aerial imagery of the six dolphins at DQO was collected by two UAS platforms during June 2019. However, individual A was sampled by one platform only due to weather (Table A2). A DJI Inspire‐2 quadcopter and an Aerial Imaging Solutions APH‐22 hexacopter were used to collect aerial imagery. The Inspire‐2 was equipped with a DJI Zenmuse X5s digital camera (20.8‐megapixel, Micro Four Thirds format; calibrated following Dawson et al. (2017)) with an Olympus M.Zuiko 25 mm f/1.8 lens. The APH‐22 was equipped with an Olympus E‐PM2 digital camera (16.1‐megapixel, Micro Four Thirds format), also with an Olympus M.Zuiko 25 mm f/1.8 lens. A LightWare SF11/C laser altimeter (Dawson et al., 2017) was attached to both platforms, providing an accuracy of 0.1 m and resolution of 1 cm. Despite the precision, some inaccurate altitude readings were recorded. To correct these errors, a custom‐made smoother was applied to the original data. The Inspire‐2 recorded videos in 4 k resolution (3840 × 2150 pixels), while photographs (4608 × 3456 pixels) were taken with the APH‐22. Consecutive flights using both platforms (*n* = 24 flights in total) were conducted at five altitudes (16, 20, 30, 40, and 50 m).

**FIGURE 2:**
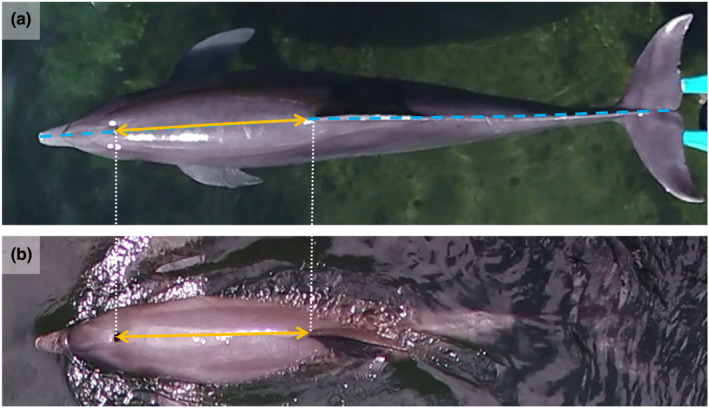
UAS video‐still images of an individual bottlenose dolphin at Dolphin Quest Oʻahu (HI, USA). UAS measurements were collected for (a) stationary and (b) surfacing animals while swimming. UAS measurements consisted of the TL (i.e., tip of the rostrum to the notch in the flukes; dashed blue line shown in (a), and BHDF (i.e., the center of the blowhole to the anterior insertion of the dorsal fin; orange arrows shown in (a) and (b)). BHDF, blowhole to dorsal fin distance; TL, total length; UAS, Unoccupied Aerial System.

Dolphins were sampled under two scenarios: stationary and positioned flat and straight in the water (Figure 2a) and with the slight arching that occurs when surfacing naturally while swimming (Figure 2b). Stationary animals were supported by husbandry staff under the caudal region to maintain the body straight and the fluke flat (Figure 2a). Photogrammetry of stationary behaviors was collected to compare UAS measurements of TL and BHDF (Figure 2) with the respective physical measurements (Figure 1a).

For each UAS platform, a target of three images was selected per individual, altitude, and behavior (i.e., stationary and surfacing) combination. For the Inspire 2, images were extracted using VLC Media Player Software (VideoLAN). For surfacing dolphins, video stills and photographs were selected when both the blowhole and dorsal fin insertion were visible and when the individual's body was as straight and horizontal as possible (i.e., minimal body arch). Available images of sufficient quality varied by platform (Table A2). In total, 144 video stills (75 stationary and 69 surfacing) from the Inspire‐2 and 127 photographs (65 stationary and 62 surfacing) of sufficient quality were used to compare the platforms (Table A2). Due to weather or the lack of images of sufficient quality, individual A (APH‐22) and individual F were removed from the analyses (both platforms).

Images from each platform were processed by two independent observers using an updated version of the Graphical User Interface (GUI) described in Dawson et al. (2017). Image processing consisted of measuring TL and BHDF for stationary animals and measuring BHDF for surfacing animals. Using a Wilcoxon test, no significant differences in accuracy (Table A4) were found between observers for the measurements made for each platform (Table A3). However, there were significant differences between observers across platforms (Table A4), with the APH‐22 observers producing more precise measurements of TL and more accurate measurements of BHDF (Table A3). The sample of APH‐22 images was smaller than that of the Inspire‐2 because suitable images were more likely to be obtained from the Inspire‐2 video footage than the APH‐22 photographs (Table A2). Given the inter‐observer reliability and greater efficiency of the Inspire‐2, only Observer 1's measurements of the Inspire‐2 video‐still images were used for the remainder of the study.

### 2.3 Calculating the error of UAS measurements

**FIGURE A2:**
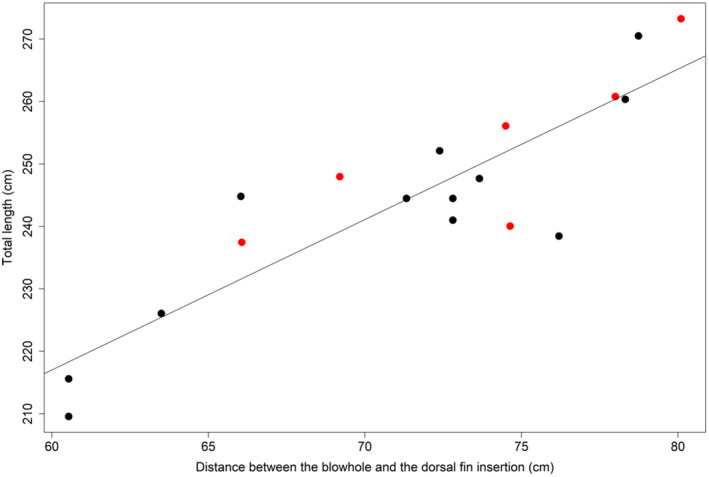
Relationship between the physical TL and physical BHDF for bottlenose dolphins under human care (Dolphin Quest, HI, USA). Red dots represent dolphins measured at the Dolphin Quest Oʻahu facility (*n* = 6), while black dots represent dolphins measured at the Dolphin Quest Hawaiʻi facility (*n* = 12). The black line depicts the BHDF to TL relationship (*n* = 18, *R* ^2^ = .76, y=2.408x+72.521). BHDF, blowhole to dorsal fin distance; TL, total length.

Using a frequentist approach, UAS photogrammetry error was calculated as the difference between the physical and UAS measurements of TL and BHDF from five stationary animals at DQO. We quantified the relationship between physical measurements of TL (cm) and BHDF (cm, a proxy for TL) of the 18 Dolphin Quest animals (Figure A2) and tested three models (ratio of BHDF/TL, linear, log‐transformed linear) to estimate TL via BHDF (see Methods A1). To first evaluate the performance of these models (Methods A1), each model's coefficients were used to separately estimate TL based on physical measurements of BHDF (BHDF_Physical_) from the five stationary dolphins at DQO. Since the models performed well on physical measurements (Table 2), we then used them to estimate TL from UAS‐measured BHDF (BHDF_UAS_) for five surfacing animals (see Methods A1). The error (± standard deviation, SD) in estimating TL from BHDF for the surfacing animals was calculated for each model (see Methods A2). Based on the model performances (Table 2), the log‐transformed linear model was considered the best model for use in subsequent analyses. Table 1 summarizes the data sources, data types, and associated analyses for this and the following section.

**TABLE 1:** Summary of sample sizes, facility versus community, data collected (i.e., physical vs. UAS‐measured), and analyses performed.

Location (facility or data source)	Number of dolphins	Type of data collected	Modeling the relationship between TL and BHDF	Estimating TL from UAS‐measured BHDF	Testing accuracy of age classifiers using TL estimates and BHDF
Oʻahu (Dolphin Quest, HI, USA)	5	UAS measurements	Yes	Yes	No
Oʻahu + Hawaiʻi (Dolphin Quest, HI, USA)	18 (6 + 12)	Physical measurements	Yes	No	No
Sarasota (SDRP, FL, USA)	268	Physical measurements	No	No	Yes

Abbreviations: BHDF, blowhole to dorsal fin distance; TL, total length; UAS, Unoccupied Aerial System.

### 2.4 Testing the performance of UAS estimates to infer age‐class

To test the feasibility of assigning individuals to age‐class bins using UAS estimates of TL (from BHDF), we employed a long‐term morphometric dataset of bottlenose dolphins from the SDRP. Since 1984, the SDRP has been conducting periodic catch‐and‐release of individuals for life history studies and health assessment (Wells, 2009; Wells et al., 2004). During these assessments, physical measurements of dolphins were obtained, including TL and other measurements we used to derive BHDF (Figure 1b). In total, 742 health assessments were made of 263 unique individuals of both sexes during 1984–2019. We used the following information from the SDRP dataset: age (years, either empirical DOB from observations of the animal and its identifiable mother, or, if the DOB was unknown, an estimate from growth layer groups in a tooth extracted under local anesthesia (Hohn et al., 1989)); TL (cm), distance between the tip of the rostrum and the center of the caudal edge of the blowhole (cm); and distance between the tip of the rostrum and the anterior insertion of the dorsal fin (cm). BHDF for each animal in the SDRP dataset was calculated by subtracting the second‐to‐last measurement from the last. While these BHDF measurements include the diameter of the blowhole, we assumed they did not differ significantly from BHDF measurements that terminate in the center of the blowhole (difference of approximately 1 cm, F.V. personal observation).

We followed a frequentist approach to test the performance of UAS photogrammetry in inferring age‐class. New sets of BHDF measurements and associated TL estimates were simulated for the SDRP long‐term morphometric dataset by applying the UAS errors (±SD) in estimating TL using BHDF of surfacing animals. However, because of the limited sample size (*n* = 5) and overall above‐average TL/BHDF relationship resulting from the physical measurements of the DQO individuals compared with the DQH individuals (Figure A2), the error of the UAS‐simulated measurements was set to 0 plus the calculated SD. This prevented from overestimating the size of the SDRP dolphins. First, the UAS‐simulated measurements of BHDF were calculated following:

(1) $${\text{BHDF}}_{\mathrm{UA}S_{\mathrm{sim}}}={\text{BHDF}}_{\mathrm{SDR}P_{\text{physical}}}*\left(1+N\left(n,D_{e_{\text{BHDF}}},D_{sd_{\text{BHDF}}}\right)\right)$$

where ${\text{BHDF}}_{\mathrm{UAS}\_\mathrm{sim}}$ is the UAS‐simulated BHDF measurement, ${\text{BHDF}}_{\text{SDRP}\_\text{physical}}$ is the physical measurement of BHDF (from the SDRP dataset), *N* is the normal distribution, *n* is the number of SDRP individuals, *D*_e*_*BHDF_ is the UAS error calculated with BHDF of surfacing animals, and *D*_sd_BHDF_ is the UAS SD for the UAS‐measurement error.

Similarly, UAS‐simulated TL estimates based on ${\text{BHDF}}_{\mathrm{UAS}\_\mathrm{sim}}$ were calculated:

(2) $${\mathrm{TL}}_{\mathrm{UA}S_{\mathrm{sim}}}={\mathrm{TL}}_{\mathrm{Est}.\log.\text{linear}}*\left(1+N\left(n,D_{e},D_{\mathrm{sd}}\right)\right)$$

where ${\mathrm{TL}}_{\mathrm{UAS}\_\mathrm{sim}}$ is the UAS‐simulated TL, ${\mathrm{TL}}_{\mathrm{Est}.\log.\text{linear}}$ is the TL estimated by the log‐transformed linear model (Equation S4) using ${\text{BHDF}}_{\mathrm{UAS}\_\mathrm{sim}}$, *N* is the normal distribution, *n* is the number of SDRP individuals, *D*_e_ is the UAS error with estimates of TL via BHDF of surfacing animals, and *D*_sd_ is the SD for the UAS‐measurement error.

Next, age classifiers were created to determine the proportion of SDRP individuals correctly assigned to their actual (or known) age‐class bins based on their UAS‐simulated BHDF measurements and TL estimates. We defined five classification scenarios ranging from narrow to broad age‐class bins; age spans (“X–Y”, “X+”, in years) within each scenario read as, respectively, “greater than or equal to X and less than Y years old” or “greater than or equal to X”. These scenarios were selected to test the effect of age‐bin widths (narrow vs. broad) on the age classification, based around the reproductive status of the animals. Scenario A has seven age‐class bins (“0–3”, “3–7”, “7–15”, “15–25”, “25–35”, “35–40”, and “40+”), Scenario B has six age‐class bins (“0–2”, “2–4”, “4–6”, “6–8”, “8–10”, and “10+”), Scenario C has four age‐class bins (“0–3”, “3–8”, “8–15”, and “15+”), Scenario D has three age‐class bins (“0–2”, “2–10”, and “10+”), and Scenario E has two age‐class bins (“0–10” and “10+”). Scenario D was designed following the age classification from Herrman et al. (2020) for the dolphins of the Sarasota community (i.e., calves, juveniles, and adults). For each scenario, the mean (±SD), minimum, and maximum values of physical BHDF and TL were calculated for every age‐class bin using the SDRP dataset. These length distributions were subsequently used to calculate the probabilities of assigning each individual across all age‐classes using ${\text{BHDF}}_{\mathrm{UAS}\_\mathrm{sim}}$ and ${\mathrm{TL}}_{\mathrm{UAS}\_\mathrm{sim}}$. The performance of each age classifier was determined by calculating the proportions of time (in %) SDRP dolphins were correctly assigned to their actual age‐class Finally, we simulated how young individuals (<10 years) were classified across all age‐classes under Scenario B. This allowed us to better understand whether correctly classifying younger individuals was possible, and how these animals were assigned based on UAS length estimates. Additionally, this allowed us to visualize where individuals were assigned when not correctly classified. Results were averaged over 1000 simulations. Testing both UAS‐simulated estimates of BHDF measurements and TL estimates allowed us to determine whether UAS‐simulated BHDF could be used alone to assign an age‐class to individuals, rather than estimating TL from the UAS‐simulated BHDF.

## 3 RESULTS

### 3.1 Physical length measurements

Physical measurements of TL for stationary dolphins averaged 252.6 ± 1.1 cm (mean ± SD, 235.0–274.3, *n* = 6) and 241.3 ± 2.2 cm (212.1–272.6, *n* = 12) at DQO and DQH, respectively. Physical measurements of BHDF for stationary dolphins averaged 73.6 ± 1.0 cm (66.1–80.1) and 70.6 ± 0.9 cm (60.5–78.7, *n* = 263) at DQO and DQH, respectively. Similarly, true TL of Sarasota dolphins averaged 234.7 ± 26.1 cm (166–285) and true BHDF averaged BHDF 71.7 ± 8.6 cm (46–107.5).

### 3.2 Calculating the error between physical versus UAS measurements

**FIGURE 3:**
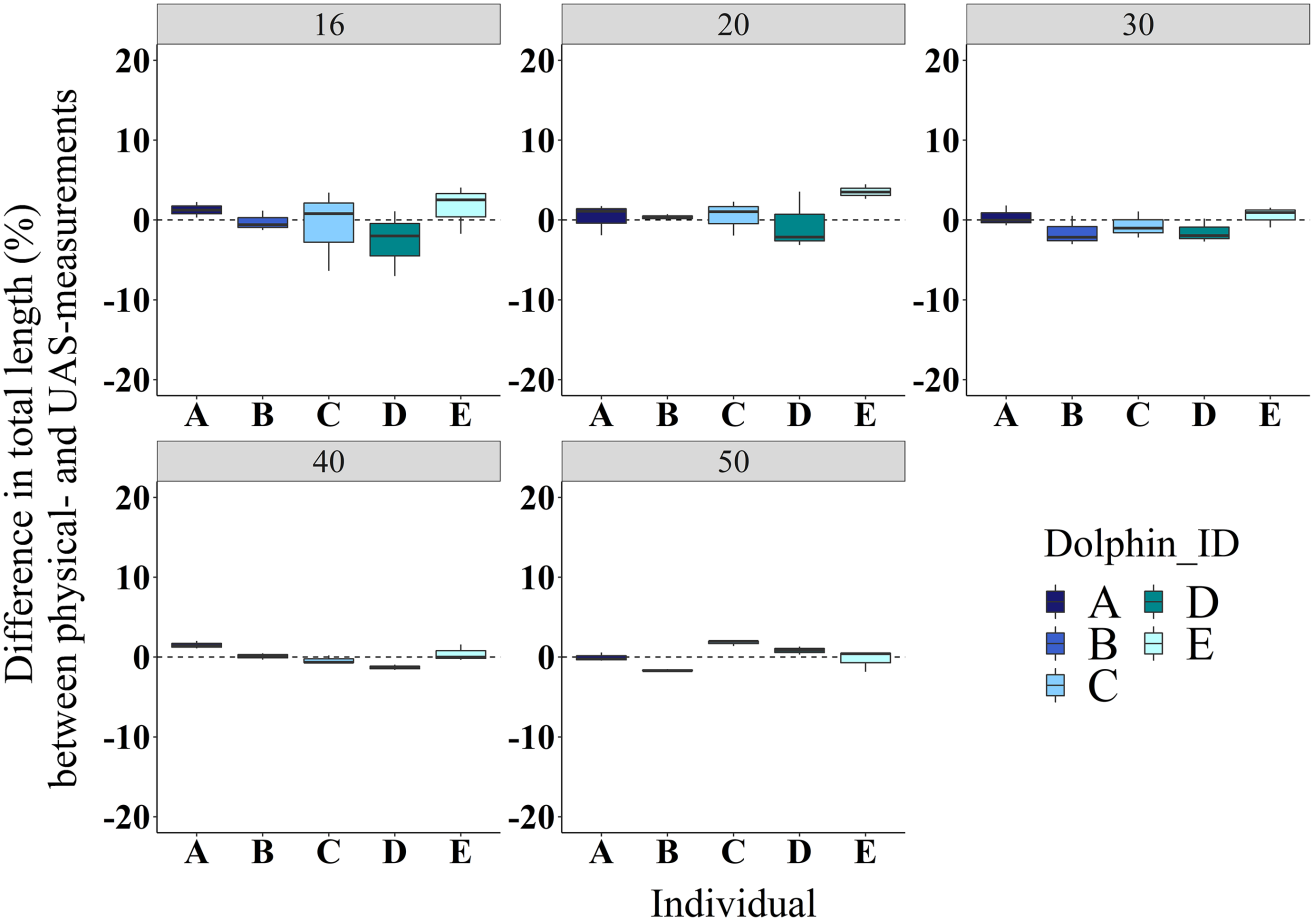
Mean differences (%) in total length between physical measurements and UAS estimates of five stationary bottlenose dolphins. Errors are represented for each altitude (m, gray header) and individuals are color‐coded (a–e). The dashed line indicates zero difference. The horizontal bold line represents the median value, and the whiskers represent the upper and lower 25% of values. Sample sizes can be found in Table A2. UAS, Unoccupied Aerial System.

**FIGURE A3:**
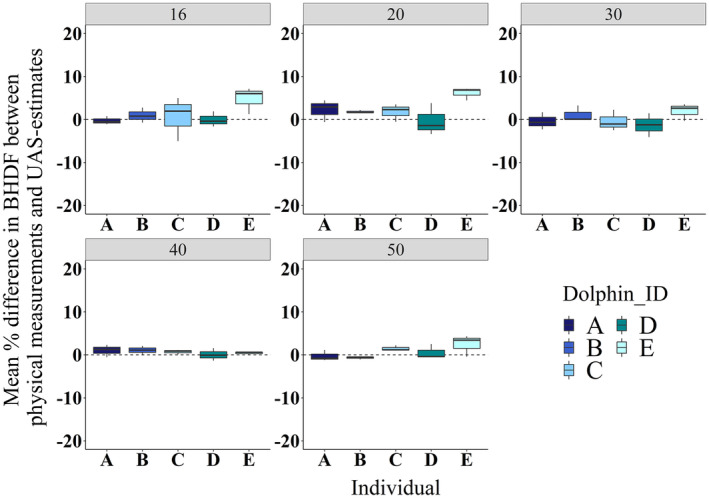
Mean differences (%) in BHDF between physical measurements and UAS‐measurements of five stationary bottlenose dolphins. Errors are represented for each altitude (m, grey header) and individuals are color‐coded (A–E). The dashed line indicates zero difference, and error bars represent 95% confidence intervals. BHDF, blowhole to dorsal fin distance; UAS, Unoccupied Aerial System.

The average difference between the TL measurements of stationary dolphins by physical and UAS methods was 0.1 ± 1.3% (mean ± SE) across all five altitudes (Figure 3). The levels of accuracy of UAS measurements were similar regardless of altitude, suggesting that sampling can be successfully conducted between 16 and 50 m. However, precision in the measurements was better using images collected from 40 and 50 m altitudes (Figure 3). Similarly, the difference between BHDF measurements of stationary dolphins by physical and UAS methods was 1 ± 2.4% (Figure A3).

**FIGURE 4:**
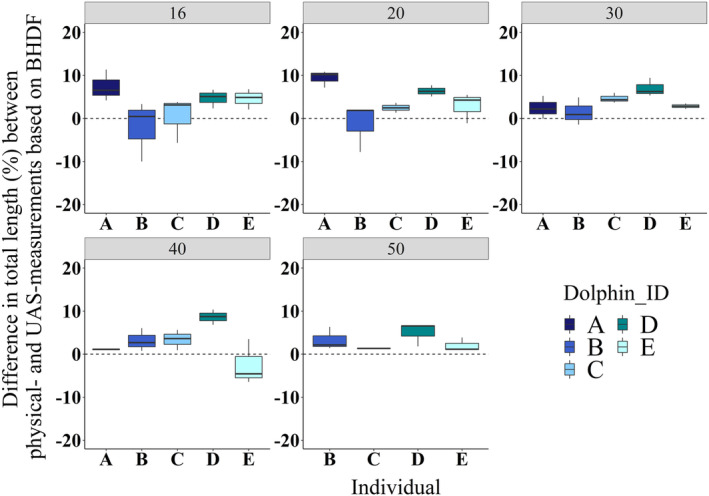
Mean differences (%) in total length between physical measurements and UAS estimates from BHDF of five surfacing bottlenose dolphins. Errors are represented for each altitude (*m*, gray header) and individuals are color‐coded (a–e). The dashed line represents zero difference. The horizontal bold line represents the median value, and the whiskers represent the upper and lower 25% of values. Sample sizes can be found in Table A2. BHDF, blowhole to dorsal fin distance; UAS, Unoccupied Aerial System.

Across all altitudes, dolphin TL estimated through UAS photogrammetry using “surfacing” BHDF (i.e., ${\mathrm{TL}}_{\mathrm{Est}.\log.\text{linear}}$; Equation S4) were overestimated by 3.3 ± 3.1% compared with their corresponding physical measurements (Figure 4). All altitudes provided similar levels of accuracy, although greater precision was achieved for the three highest altitudes (Figure 4).

### 3.3 Estimating TL using UAS measurements of BHDF

The ratio between physical measurements of BHDF and TL (Equation S1) was 29.2, with BHDF representing approximately 30% of TL. Applying this ratio (Equation S2), TL was underestimated by 0.2 ± 4.2% using physical measurements of BHDF. Using a linear relationship between TL and BHDF (*p*‐value <.001, *R* ^2^ = .76, Figure A2), TL estimates based on physical measurements of BHDF were underestimated by 0.9 ± 3.2% and 1.0 ± 3.2% with the linear (Equation S3) and log‐transformed linear models (Equation S4). These results indicated that these models can be used to accurately estimate TL via BHDF (Table 2). Therefore, we used the same models to estimate TL of surfacing animals using UAS measurements of their BHDF. UAS‐estimated TL were overestimated by 6.8 ± 3.8%, 3.4 ± 3.1%, and 3.3 ± 3.1% with the ratio, linear, and log‐transformed linear models, respectively (Table 2).

**TABLE 2:** Mean error (% difference ± SE) between physical TL and estimated TL for five bottlenose dolphins.

Equation	Mean TL difference (%)	
	Physically measured BHDF	UAS‐measured BHDF
${\mathrm{TL}}_{\mathrm{Est}.\text{ratio}}$ (Equation S2)	−0.2 ± 4.2	6.8 ± 3.8
${\mathrm{TL}}_{\mathrm{Est}.\text{linear}}$ (Equation S3)	−0.9 ± 3.2	3.4 ± 3.1
${\mathrm{TL}}_{\mathrm{Est}.\log.\text{linear}}$ (Equation S4)	−1.0 ± 3.2	3.3 ± 3.1

*Note*: TL was estimated (a) using average physically measured BHDF, and (b) using average UAS‐measured BHDF for surfacing animals across five altitudes. ${\mathrm{TL}}_{\mathrm{Est}.\text{ratio}}$ is the estimated TL using the ratio between TL and BHDF, ${\mathrm{TL}}_{\mathrm{Est}.\text{linear}}$ is the estimated TL using a linear model, and ${\mathrm{TL}}_{\mathrm{Est}.\log.\text{linear}}$ is the estimated TL using a log‐transformed linear model.

Abbreviations: BHDF, blowhole to dorsal fin distance; TL, total length; UAS, Unoccupied Aerial System.

### 3.4 Testing the performance of the age classifiers using UAS‐simulated TL estimates

Mean age‐classifier performance increased from 34.6% to 79.8% of correctly assigned individuals as the number of age‐class bins was reduced (Table 3). Additionally, classifier performance was nearly equivalent between UAS‐simulated BHDF measurements and TL estimates, with the BHDF method performing better for the youngest age‐class bins (0–3 and 0–2 years, Table 3). Across all scenarios, performance was best in the youngest age‐class bins. Overall, performance was best when using three age‐class bins (around 72% for both TL and BHDF methods, Scenario D) or two age‐class bins (79.8% and 79.1% for TL and BHDF methods respectively, Scenario E).

**TABLE 3:** Performance of the age classifiers using UAS‐simulated BHDF measurements and associated TL estimates on the Sarasota dolphin dataset.

Age‐class bin scenario	Age‐class bins	0–3 (*n* = 107)	3–7 (*n* = 177)	7–15 (*n* = 180)	15–25 (*n* = 149)	25–35 (*n* = 81)	35–40 (*n* = 25)	40+ (*n* = 23)	Mean (*n* = 742)
A	Accuracy TL (%)	65.9	50.0	25.6	17.4	19.7	19.6	21.3	34.6
	Accuracy BHDF (%)	71.1	37.3	21.4	20.1	20.1	21.6	22.4	32.1

	Age‐class bins	0–2 (*n* = 27)	2–4 (*n* = 128)	4–6 (*n* = 91)	6–8 (*n* = 73)	8–10 (*n* = 42)	10+ (*n* = 381)	Mean (*n* = 742)	
B	Accuracy TL (%)	57.4	35.7	29.1	30.3	27.8	49.0	41.5	
	Accuracy BHDF (%)	69.0	35.5	26.7	25.6	22.9	52.9	42.9	

	Age‐class bins	0–3 (*n* = 107)	3–7 (*n* = 212)	7–15 (*n* = 145)	15+ (*n* = 278)	Mean (*n* = 742)			
C	Accuracy TL (%)	64.2	53.0	42.2	50.5	51.1			
	Accuracy BHDF (%)	72.5	43.1	36.7	54.8	50.2			

	Age‐class bins	0–2 (*n* = 27)	2–10 (*n* = 334)	10+ (*n* = 381)	Mean (*n* = 742)				
D	Accuracy TL (%)	77.1	70.3	75.1	73.0				
	Accuracy BHDF (%)	83.8	62.2	77.8	71.0				

	Age‐class bins	0–10 (*n* = 361)	10+ (*n* = 381)	Mean (*n* = 742)					
E	Accuracy TL (%)	83.8	76.1	79.8					
	Accuracy BHDF (%)	79.2	78.9	79.1					

*Note*: Results are categorized per age‐class bin Scenarios A, B, C, D, and E. Age‐class bins are expressed in years, the performance is expressed in percentages, and *n* represents the number of individuals in each age‐class.

Abbreviations: BHDF, blowhole to dorsal fin distance; TL, total length; UAS, Unoccupied Aerial System.

**FIGURE 5:**
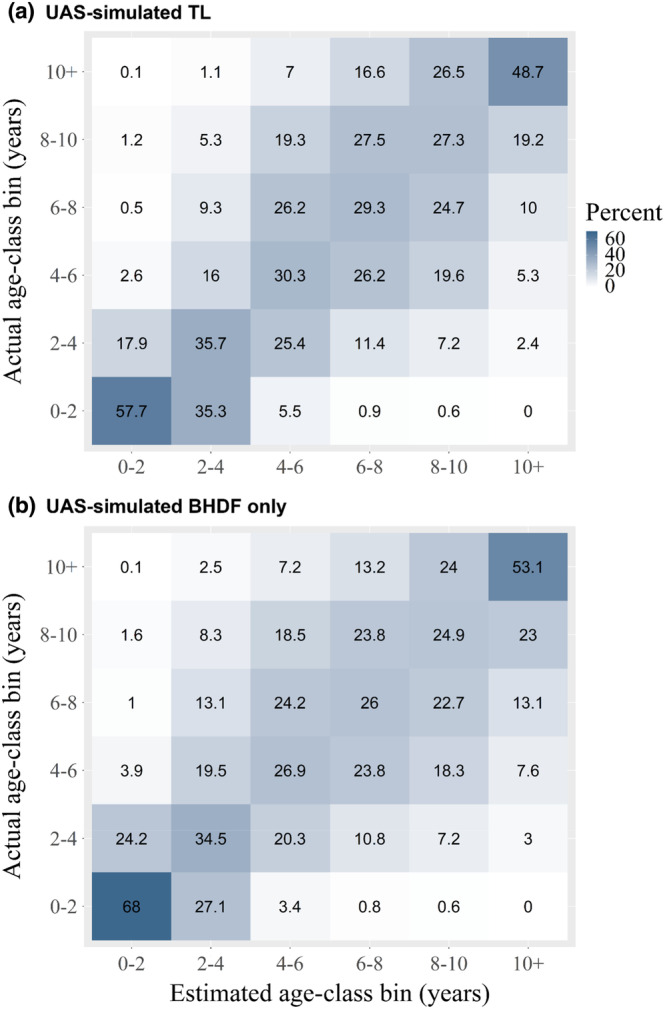
Mean proportions of correctly assigning age‐class bins to individuals under Scenario B using (a) UAS‐simulated TL from UAS‐simulated BHDF measurements and (b) UAS‐simulated BHDF measurements only. Results were averaged over 1000 simulations. Darker cells indicate a better performance; age‐class bins are expressed in years and the probability is expressed in percentage. The sample size for each age‐class bin is listed in Table 4. A probability distribution in assigning individuals to correct age‐class bins for both UAS‐simulated BHDF and TL methods can be found in the Supplementary Materials (Figure A4). BHDF, blowhole to dorsal fin distance; TL, total length; UAS, Unoccupied Aerial System.

Finally, under Scenario B, we quantified where individuals were classified when not assigned to their correct age‐class using UAS‐simulated TL estimates (Figure 5a) and UAS‐simulated measurements of BHDF only (Figure 5b). Overall, 72.5%–93% of the individuals were correctly classified within two age‐class bins (one age‐class bin younger and older) of their actual age‐class bin (Table 4a). Similar results were obtained when using UAS‐simulated BHDF measurements only (Figure 5b; Table 4b).

**TABLE 4:** Mean proportions of individuals correctly assigned within two age‐class bins under Scenario B and simulated (a) UAS‐estimated TL via BHDF measurements and (b) UAS‐measured BHDF only.

	Age‐class Bins (years)	Probability (%) of correct assignment in the following age‐class bins					
	_Actual_^Estimated^	0–2	2–4	4–6	6–8	8–10	10+
(A) TL based on BHDF	**0–2** (*n* = 27)	**93**		5.5	0.9	0.6	0
	**2–4** (*n* = 128)	**79**			11.5	7.3	2.4
	**4–6** (*n* = 91)	2.6	**72.5**			19.7	5.2
	**6–8** (*n* = 73)	0.5	9.3	**80.2**			10.0
	**8–10** (*n* = 42)	1.2	5.3	19.3	**74**		
	**10+** (*n* = 381)	0.1	1.1	7	16.6	**75.2**	

	_Actual_^Estimated^	0–2	2–4	4–6	6–8	8–10	10+
(B) BHDF	**0–2** (*n* = 27)	**95.1**		3.4	0.8	0.6	0
	**2–4** (*n* = 128)	**79**			10.8	7.2	3.0
	**4–6** (*n* = 91)	3.9	**70.2**			18.3	7.5
	**6–8** (*n* = 73)	0.9	13.0	**72.9**			13.1
	**8–10** (*n* = 42)	1.6	8.3	18.4	**71.7**		
	**10+** (*n* = 381)	0.1	2.5	7.2	13.2	**77.1**	

*Note*: Results were averaged over 1000 simulations. Shaded fused cells and bold numbers indicate the probability of assigning individuals to within two age‐class bins of the actual age‐class bin tested. Numbers in brackets represent the sample size per actual age‐class bin.

Abbreviations: BHDF, blowhole to dorsal fin distance; TL, total length; UAS, Unoccupied Aerial System.

## 4 DISCUSSION

Early detection of changes in vital rates of free‐ranging delphinids due to environmental and anthropogenic stressors is needed to better forecast changes in population dynamics. Despite some caveats, we successfully tested and simulated a new approach using UAS photogrammetry to assess the population age structure of bottlenose dolphins, demonstrating the utility of UAS photogrammetry for quantifying age‐class structure in free‐ranging delphinid populations, which, in turn, can facilitate the detection of early signs of population changes.

Our study was divided into two components. The first aimed to ground‐truth the precision and accuracy of UAS photogrammetry measurements by comparing physical to UAS‐measured distances of TL and BHDF of bottlenose dolphins under human care. TL estimates of surfacing dolphins from UAS‐measured BHDF of surfacing dolphins were overestimated by 3.3 ± 3.1% (Table 2) using log‐transformed linear models (Methods S1). The second component aimed to evaluate our ability to infer age‐class using TL estimates (based on UAS measurements of BHDF) or UAS measurements of BHDF alone and then assess the age structure of a free‐ranging community of bottlenose dolphins (Figure 1). Our approach performed best (~80% and ~72%) when classifying individuals into age‐class bins, especially into two (“0–10” and “10+”) or three (“0–2”, “2–10”, and “10+”) bins, respectively (Table 3).

### 4.1 Accounting for sources of error between physical measurements and UAS estimates

The precision and accuracy of UAS‐estimated TL resulting from this study compare favorably with other photogrammetric methods used to measure free‐ranging marine mammals. UAS photogrammetric methods overestimated the TL of leopard seals (*Hydrurga leptonyx*) by ~2.0% (Krause et al., 2017), Australian snubfin dolphins (*Orcaella heinsohni*), and humpback dolphins (*Sousa sahulensis*) by ~3.0% (Christie et al., 2021), and manatees (*Trichechus manatus manatus*) by ~8.0% (Ramos et al., 2022). Similarly, using stereo‐laser photogrammetry, TL estimates of bottlenose dolphins based on BHDF were overestimated by 1.4% (Cheney et al., 2018) and 1.9% (van Aswegen et al., 2019).

A negligible difference was documented in inter‐observer (1 and 2) measurements of TL and BHDF estimates for the Inspire 2, despite a significant difference across observers and platform (Tables A3 and A4). However, various sources of error could influence the accuracy and the precision of UAS photogrammetry. The most notable source of error was inaccurate readings of UAS altitude at the time of image collection (Perryman & Lynn, 1993), as precise altitude readings are required to convert image pixel lengths to absolute measurements (Dawson et al., 2017). The location and positioning of the altimeter custom‐mounted on the body frame of the Inspire‐2 (instead of the camera gimbal) may have altered its altitude readings and resulted in bias. That is, the altimeter on the Inspire‐2 points 90° down in relation to the UAS body frame, whereas the altimeter on the APH‐22 was mounted on the camera gimbal and points ~90° down in relation to the ground. However, altitude corrections were applied to account for pitch and roll of the Inspire‐2 (Christiansen et al., 2018). Shadows and/or sun reflection on the animal's body can obscure the identification of the blowhole and the insertion of the dorsal fin, thus potentially biasing our TL estimates. Our results suggested the curvature of the dolphin's body as a bias, also supported by Jaquet (2006). Additional sources of error include angle of the camera and lens distortion (Burnett et al., 2019), and water distortion (Dittmann & Slooten, 2016). Finally, an innovative Bayesian approach for propagating uncertainty in laser readings down to parameters of interest (i.e., TL, BHDF, age‐class) was developed (Bierlich, Hewitt, et al., 2021; Bierlich, Schick, et al., 2021). Our frequentist approach addressed this uncertainty by smoothing the raw altitude data to account for laser reading uncertainty. However, this smoothing may underestimate the uncertainty in the measurements of TL (and slightly narrow down the error bars around these), and age classification. Future research should explore how the two different approaches to addressing uncertainty vary in age‐class probabilities. Despite these possible sources of errors, our findings support the accurate performance of UAS photogrammetry to infer TL based on BHDF measurements of surfacing animals.

Three modeling approaches were tested to estimate the TL of surfacing bottlenose dolphins from UAS‐measured BHDF. Deriving TL using the TL/BHDF ratio provided the least accurate estimates. Results from the two other methods were similar, albeit with slightly better estimates using the log‐transformed linear model. However, some limitations may arise from a log transformation, as it may make the data more variable and skewed (Feng et al., 2014). While strong, the strength of the relationship between TL and BHDF for the 18 bottlenose dolphins at DQO and DQH (*R* ^2^ = .76) was less than those documented for free‐ranging bottlenose dolphins (*R* ^2^ = .96, *n* = 11—Cheney et al., 2018; and *R* ^2^ = .99, *n* = 129—van Aswegen et al., 2019) and the Sarasota bottlenose dolphin community (*R* ^2^ = .86, *n* = 282—unpublished results provided by R.W.). We believe this may be due to our small sample size (*n* = 18). Overall, despite our small sample size, we confirmed the high accuracy of UAS photogrammetry in obtaining accurate morphometric measurements of dolphins. However, the relationship between TL and BHDF may differ between populations. In Australia, stereo‐laser photogrammetry demonstrated that the growth rate and individual body lengths differed between two Australian populations of Indo‐Pacific bottlenose dolphins (*Tursiops aduncus*), where individuals in temperate waters were significantly longer (~30%) than their counterparts inhabiting sub‐tropical waters (van Aswegen et al., 2019).

### 4.2 Quantifying the age structure of the Sarasota dolphin community

Assigning individuals to age‐classes via UAS photogrammetry using TL (via BHDF) or BHDF is a promising approach to inform population assessments when age‐length growth curves are available for the study population. We obtained high classification scores when predicting the age of individuals to within two age‐class bins of the actual age‐class (Figure 5) and demonstrated that a narrower age‐class bin width is less likely to correctly age‐classify older animals (Table 3, Scenarios A and C) because of overlapping length distributions. Our findings highlight the importance of defining appropriate age‐class bins for the study population. In this study, classifying individuals into three or two age‐classes performed best. Our findings compared with those by Cheney et al. (2022), who correctly age‐classified ~66% of the bottlenose dolphins they sampled (*n* = 54) in Scotland using five age‐classes. However, they concluded that TL determined via UAS photogrammetry was not fully reliable to correctly age‐classify individuals.

In Sarasota, the age‐length growth curve for the bottlenose dolphin reaches a plateau at 10–15 years (Read et al., 1993). Such a growth pattern may explain the difficulty in accurately estimating ages of individuals (via TL) for a range of ages that do not significantly differ in length (e.g., 10+ years old). Nonetheless, we demonstrated an acceptable age classification of younger dolphins (<10 years) independent of age‐class bin width. In this study, the age classifier performance was better when fewer and broader age‐class bins were used for assigning individuals based on UAS‐simulated BHDF measurements and TL estimates, and was best when two age‐classes were used (“0–10” and “10+” years, Table 3).

Similar accuracies were obtained whether using UAS‐simulated BHDF measurements or associated TL estimates when age‐classifying individuals (Table 3). Since UAS‐measured BHDF alone seems promising to quantify the age structure of free‐ranging delphinid populations, there is potential for this method to be used for populations with little to no readily available demographic information. Although assumptions would have to be made about their age‐length growth curves, large offshore populations may greatly benefit from this method since UAS photogrammetry could allow for efficient sampling of large groups.

### 4.3 Age structure and conservation: applicability

Data‐informed population models are required for the sustainable management of wildlife populations (Crouse et al., 1987; Morris et al., 2011). The age structure of individuals within a population is often at the center of these models, as other parameters such as individual growth (Clark et al., 2000) and survival and reproductive rates (Barlow & Boveng, 1991; Loison et al., 1999) vary by age. The ability to assign individuals to age‐classes not only benefits the study of populations (Crouse et al., 1987; Holmes et al., 2007; Slooten & Lad, 1991), but it also enables the quantification of changes in survival and other parameters within and across age‐classes (Holmes & York, 2003). For instance, survival through the first winter was strongly related to the length of bottlenose dolphin calves in Moray Firth, Scotland (Cheney et al., 2018).

Unstable demographic structure (e.g., an unbalanced age structure or sex ratio) has significant implications for population dynamics (Jackson et al., 2020). In Moray Firth, an increase in juvenile/adult bottlenose dolphin survival over a 25‐year timespan was most likely caused by a 45% decrease in juvenile mortality rate (Civil et al., 2018). Booth et al. (2020) evaluated the utility of the ratio of calves to mature females and the proportion of non‐adults in the population, while simulating the likely effectiveness of a monitoring program on a declining population of 40% or more by the end of the disturbance period. They highlighted the importance of the proportion of immature individuals in a population, for which a proportion of 20% or more correctly identified 81% of all declines in year 5 (Booth et al., 2020). These results suggested that the ratio of immature animals in the population may help with forecasting a potential population reduction. UAS photogrammetry may facilitate the assessment of the proportion of individuals within specific age‐classes within a population over time and may help detect or forecast potential changes in a population.

Our method focused on a bottlenose dolphin population; however, it can be applied to other delphinid species of interest. Spinner dolphins (*Stenella longirostris*) in the main Hawaiian Islands have become a management priority since 2005^1^ ^,^ ^2^ Increasing tourism activities in the resting areas used by these dolphins (Heenehan et al., 2017; Tyne et al., 2018) have raised concerns for the long‐term viability of island‐associated populations. Boat‐based photo‐identification surveys designed to estimate the abundance of spinner dolphins in Kona, Hawaiʻi, revealed a lower population size (*n* = 631, 95% CI 524–761; Tyne et al., 2014) than previously estimated (960 and 1001; Norris et al., 1994; Östman‐Lind et al., 2004, respectively). However, 9 years of similar surveys would be required to detect a 37% decline in this spinner dolphin stock (Tyne et al., 2016). Designing surveys to determine the age structure of the groups encountered may facilitate understanding the overall age structure of the population studied. Faster detections of population changes may be facilitated using UAS photogrammetry as a practical and efficient tool to monitor the age structure of cetacean populations.

## 5 CONCLUSION

Our study demonstrates the use of UAS photogrammetry as a promising and reliable tool for monitoring the age structure of free‐ranging delphinid species. Ultimately, UAS photogrammetry has the potential to more rapidly inform management compared with traditional survey methods. This technique, as one more tool combined with other more traditional approaches, can improve precision around population demographic estimates and therefore has the potential to improve the power of population monitoring (Jacobson et al., 2020).

## AUTHOR CONTRIBUTIONS

**Fabien Vivier:** Conceptualization (equal); data curation (lead); formal analysis (lead); methodology (equal); project administration (equal); writing – original draft (lead); writing – review and editing (supporting). **Randall S. Wells:** Resources (supporting); writing – review and editing (supporting). **Marie C. Hill:** Conceptualization (supporting); data curation (supporting); methodology (equal); writing – review and editing (supporting). **Kymberly M. Yano:** Conceptualization (supporting); data curation (equal); methodology (supporting); writing – review and editing (supporting). **Amanda L. Bradford:** Conceptualization (supporting); methodology (supporting); writing – review and editing (supporting). **Eva M. Leunissen:** Software (lead); writing – review and editing (supporting). **Aude Pacini:** Conceptualization (supporting); funding acquisition (lead); methodology (supporting); project administration (equal); resources (supporting); supervision (supporting); writing – review and editing (supporting). **Cormac G. Booth:** Methodology (supporting); writing – review and editing (supporting). **Julie Rocho‐Levine:** Resources (supporting); writing – review and editing (supporting). **Jens J. Currie:** Formal analysis (supporting); writing – review and editing (supporting). **Philip T. Patton:** Writing – review and editing (equal). **Lars Bejder:** Conceptualization (equal); formal analysis (supporting); funding acquisition (equal); investigation (equal); methodology (equal); project administration (lead); resources (lead); supervision (lead); validation (lead); writing – review and editing (supporting).

## FUNDING INFORMATION

This study was funded by NOAA‐PIFSC and RCUH JIMAR (NA19NMF4720181, NA16NMF4320058), CIMAR (NA21NMF4320043), and the Office of Naval Research (N000142012624).

## CONFLICT OF INTEREST STATEMENT

The authors declare no conflict of interest.

### OPEN RESEARCH BADGES

This article has earned an Open Data badge for making publicly available the digitally‐shareable data necessary to reproduce the reported results. The data is available at https://datadryad.org/stash/dataset/doi:10.5061/dryad.d51c5b07p.

## Figures and Tables

**FIGURE A4:**
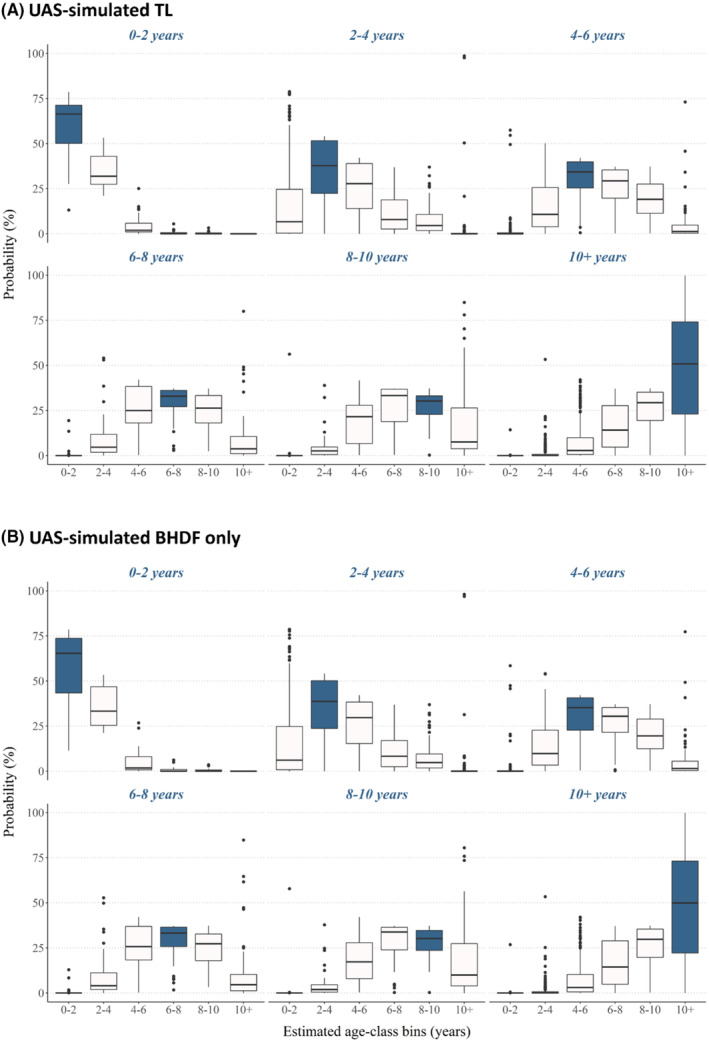
Distribution of the probabilities of age‐class bin assignment by actual age‐class bin (i.e., each subplot with a blue header). An age‐classifier was used to estimate how bottlenose dolphins were classified when not assigned to their correct age‐class under Scenario B using (a) UAS‐simulated TL from BHDF, and (b) UAS‐simulated BHDF measurements only. For each sub‐plot, the blue boxplot represents the correct assignment of the age‐class. Results were averaged over 1000 simulations. BHDF, blowhole to dorsal fin distance; TL, total length; UAS, Unoccupied Aerial System.

## Data Availability

The data collected at Dolphin Quest used to conduct the analyses reported in this manuscript are available through the Dryad Digital Repository: https://datadryad.org/stash/dataset/doi:10.5061/dryad.d51c5b07p.

## Endnotes

^1^
Federal Register/Vol. 70, No. 237 (December 12, 2005), www.govinfo.gov/content/pkg/FR‐2005‐12‐12/pdf/05‐23928.pdf.

^2^
Federal Register/Vol. 81, No. 164 (August 24, 2016), www.gpo.gov/fdsys/pkg/FR‐2016‐08‐24/pdf/2016‐20177.pdf.

## APPENDIX A

### A.1

Tables A1, A2, A3, A4 and Figures A1, A2, A3, A4.

**TABLE A1:** Summary of the abbreviations used in this manuscript and their definition.

Abbreviation	Definition
TL	Total length of dolphin. Total body length from the tip of the rostrum to the notch in the flukes
BHDF	Blowhole to dorsal fin anterior insertion. Distance between the center of the blowhole and the insertion of the dorsal fin
UAS	Unoccupied Aerial System, also referred as drone
UAS‐measured	Measured via UAS‐photogrammetry. Any length directly measured through UAS‐photogrammetry (e.g., TL and BHDF)
UAS‐estimated	Estimated via UAS‐photogrammetry. When TL is estimated based on BHDF (a proxy for TL)
UAS‐simulated	Simulated measurement based on physical measurements with the estimated UAS‐measurement error
DQO	Dolphin Quest Oʻahu. Six adult male bottlenose dolphins from this facility were physically‐ and UAS‐measured
DQH	Dolphin Quest Hawaiʻi. Six male and six female bottlenose dolphins of various ages were physically measured
SDRP	Sarasota Dolphin Research Program. SDRP's long‐term dataset (*n* = 742 physical assessments of 263 unique individuals collected from 1984 to 2019) was used to develop an age‐classifier

**TABLE A2:** Summary table of the number of images measured for each dolphin per Unoccupied Aerial System platform (UAS), altitude, and behavior (stationary/free swimming).

ID	Number of images measured per platform (stationary/free swimming)									
	Inspire‐2					APH‐22				
	16m	20m	30m	40m	50m	16m	20m	30m	40m	50m
A	3/3	3/3	3/3	3/2	3/0	–/–	–/2	–/2	–/1	–/3
B	3/3	3/3	3/3	3/3	3/3	3/–	2/3	1/3	3/3	–/–
C	3/3	3/3	3/3	3/3	3/1	3/1	3/3	3/3	2/3	3/3
D	3/3	3/3	3/3	3/3	3/3	3/3	3/3	3/3	3/3	3/3
E	3/3	3/3	3/3	3/3	3/3	3/3	3/1	3/2	3/–	3/–
F	0/0	0/0	0/0	0/0	0/0	3/2	2/2	2/2	3/2	2/3

*Note*: Shaded cells highlight individuals for which three measurements were not possible because either a flight was not conducted (–) or the flight resulted in fewer than three images of sufficient quality. Individual F was removed from the analyses for both UAS platforms, while individual A was removed from the analysis for the APH‐22 only.

**TABLE A3:** Inter‐observer difference (mean ± SE) in total length (%) between physical‐ and Unoccupied Aerial System (UAS)‐measured TL for stationary animals and BHDF for surfacing animals.

Altitude (m)	Difference between physical and UAS‐measurements (%)							
	Inspire‐2				APH‐22			
	TL (stationary)		BHDF (surfacing)		TL (stationary)		BHDF (surfacing)	
	Observer 1	Observer 2	Observer 1	Observer 2	Observer 3	Observer 4	Observer 3	Observer 4
16	−0.1 ± 1.7	−0.4 ± 2.8	5.1 ± 5.9	5.5 ± 5.5	−2.5 ± 2.4	−2.5 ± 2.5	4.3 ± 2.6	6.9 ± 3.1
20	0.8 ± 1.6	0.7 ± 1.8	6.5 ± 3.9	6.0 ± 3.0	−0.2 ± 2.0	−0.2 ± 1.8	0.7 ± 3.7	2.8 ± 3.1
30	−0.6 ± 1.0	−0.6 ± 1.6	6.2 ± 2.9	6.5 ± 3.0	−0.5 ± 1.3	−0.5 ± 1.2	1.5 ± 4.6	3.0 ± 4.7
40	0.1 ± 1.0	0.0 ± 0.5	4.9 ± 3.7	2.0 ± 4.8	−0.4 ± 1.0	0.3 ± 1.0	0.9 ± 3.3	4.1 ± 3.7
50	0.1 ± 1.3	0.1 ± 0.6	7.0 ± 3.5	7.1 ± 3.7	−0.4 ± 0.5	0.6 ± 0.6	0.5 ± 6.2	2.7 ± 5.9
Mean	0.1 ± 1.3	0.0 ± 1.5	5.9 ± 4.0	5.4 ± 4.0	−0.7 ± 1.4	−0.6 ± 1.4	1.6 ± 4.1	3.9 ± 4.1

*Note*: Observers also measured the BHDF using the same set of stationary images (results not presented but are consistent with TL results). Results are presented for each UAS‐platform.

Abbreviations: BHDF, blowhole to dorsal fin distance; TL, total length; UAS, Unoccupied Aerial System.

**TABLE A4:** Results from a paired Wilcoxon test (*p*‐values) evaluating the difference in measurement accuracy across observers and platforms (DJI Inspire‐2 and APH‐22) for stationary animals (TL) and surfacing animals (BHDF).

	Measurement accuracy (*p*‐values) between:		
	Observer 1 versus 2	Observer 3 versus 4	Observers 1 + 2 versus 3 + 4
TL (stationary)	0.10 (*V* = 0, *n* = 10) CV = 39.1	0.19 (*V* = 2, *n* = 10) CV = 18.2	<0.01 (*V* = 0, *n* = 20) CV = 161.7
BHDF (surfacing)	0.81 (*V* = 9, *n* = 10) CV = 26.3	0.06 (*V* = 0, *n* = 10) CV = 75.2	<0.01 (*V* = 55, *n* = 20) CV = 225.8

*Note*: *V* represents the measure of similarity between compared values and *n* represents the sample size. CV represents the coefficient of variation within observations (expressed in percentage).

Abbreviations: BHDF, blowhole to dorsal fin distance; TL, total length.

#### Methods A1: Estimating dolphin total length (TL) using blowhole dorsal fin distance (BHDF) proxy measurements

Three models were used to estimate TL from BHDF:

To test the performance of these models using the physical measurements of BHDF (Table 1), the parameter *BHDF*_*UAS*_ *was replaced by BHDF*_*Physical*_.

1. Ratio of BHDF/TL

The first model consisted of estimating an overall mean ratio between the mean physical BHDF and TL measurements (Ratio_BHDF/TL_) for each dolphin (*n* = 18) as follows:

(S1)
$${\text{Ratio}}_{\text{BHDF}/\mathrm{TL}}=\frac{\text{Physical BHDF}}{\text{Physical}\;\mathrm{TL}}$$

Then, TL estimates (TL_Est.ratio_, cm) for individual dolphins (*n* = 5) based on UAS‐measured BHDF (BHDF_UAS_) for surfacing animals were then calculated per altitude as follows:

(S2)
$${\mathrm{TL}}_{\mathrm{Est}.\text{ratio}}=\frac{{\text{BHDF}}_{\mathrm{UAS}}}{{\text{Ratio}}_{\text{BHDF}/\mathrm{TL}}}$$

2 Linear

Coefficients from a linear model (‘lme4’ package, R Core Team 2022) testing the relationship between physical measurements of TL and BHDF were used to estimate TL from UAS‐measurements of BHDF (cm) from surfacing animals and per altitude, as follows:

(S3)
$${\mathrm{TL}}_{\mathrm{Est}.\text{linear}}=a*{\text{BHDF}}_{\mathrm{UAS}}+b$$

where ${\mathrm{TL}}_{\mathrm{Est}.\text{linear}}$ is the TL estimated from the linear model, *a* is the slope, *b* is the y‐intercept, and BHDF_UAS_ is the UAS‐measured BHDF for surfacing animals.

3 Log‐transformed linear

The relationship between TL and BHDF was not fully isometric (i.e., linear relationship); the linear model underestimated TL for larger BHDF measurements and overestimated TL for smaller BHDF measurements. Therefore, the response and predictor variables were log‐transformed prior to using a linear model (‘lme4’ package, R Core Team 2022). TL (m) was estimated per altitude from UAS‐measurements of BHDF from surfacing animals as follows:

(S4)
$${\mathrm{TL}}_{\mathrm{Est}.\log.\text{linear}}=e^{a*\log\left({\text{BHDF}}_{\mathrm{UAS}}\right)+b}$$

where ${\mathrm{TL}}_{\mathrm{Est}.\log.\text{linear}}$ is the TL estimated from the log‐transformed linear model, *a* is the slope, *b* is the y‐intercept, and BHDF_UAS_ is the UAS‐measured BHDF for surfacing animals.

#### Methods A2: Calculating the error (%) between physical and UAS measurements

The % error in total length (TL) and blowhole to dorsal fin (BHDF) between the physical measurements and the UAS‐measurements was calculated per altitude for each dolphin as follows:

(S5)
$$D_{L}=\frac{\left(M_{\mathrm{UAS}}*100\right)}{M_{P}}-100$$

where *D*_*L*_ represents the % error in TL, *M*_UAS_ is the mean UAS‐measured TL or BHDF (cm), and *M*_P_ is the mean physical TL or BHDF (cm).
